# Information Content-Based Gene Ontology Semantic Similarity Approaches: Toward a Unified Framework Theory

**DOI:** 10.1155/2013/292063

**Published:** 2013-09-02

**Authors:** Gaston K. Mazandu, Nicola J. Mulder

**Affiliations:** Computational Biology Group, Department of Clinical Laboratory Sciences, Institute of Infectious Disease and Molecular Medicine, University of Cape Town Medical School, Observatory, Cape Town 7925, South Africa

## Abstract

Several approaches have been proposed for computing term information content (IC) and semantic similarity scores within the gene ontology (GO) directed acyclic graph (DAG). These approaches contributed to improving protein analyses at the functional level. Considering the recent proliferation of these approaches, a unified theory in a well-defined mathematical framework is necessary in order to provide a theoretical basis for validating these approaches. We review the existing IC-based ontological similarity approaches developed in the context of biomedical and bioinformatics fields to propose a general framework and unified description of all these measures. We have conducted an experimental evaluation to assess the impact of IC approaches, different normalization models, and correction factors on the performance of a functional similarity metric. Results reveal that considering only parents or only children of terms when assessing information content or semantic similarity scores negatively impacts the approach under consideration. This study produces a unified framework for current and future GO semantic similarity measures and provides theoretical basics for comparing different approaches. The experimental evaluation of different approaches based on different term information content models paves the way towards a solution to the issue of scoring a term's specificity in the GO DAG.

## 1. Introduction 

Several gene ontology (GO) semantic similarity measures have been proposed over recent years for comparing terms in the GO structure, thus allowing comparison of proteins at the functional level on the basis of their GO annotations. This has largely contributed to the efficient exploitation of the biological knowledge embedded in the GO structure. GO [1, 2] is organized as a directed acyclic graph (DAG), in which two terms are topologically linked by the relation “is_a” or “part_of” indicating that a term child is a subclass (instance) or a component of a parent term. Other relationships exist but do not impact the topology of the GO DAG as they are essentially biological. An ontology is designed to provide an explicit and semantic specification of concepts that allow knowledge about genes and their products to be described without ambiguity in a shareable and computationally accessible form, producing an efficient and standardized functional scheme. GO has been widely adopted and successfully deployed in several biological and biomedical applications, ranging from theoretical to experimental and computational biology.

In the context of high-throughput data generation, where more data are becoming available, analyzing organisms at the systems level and functional prediction for proteins of unknown function are becoming essential for better understanding of the biology of the organism. GO semantic similarity measures are tools that can be used to develop efficient and reliable strategies that allow function inference of uncharacterized proteins based on sequence comparison, microarray data analysis, or interaction networks. Very often, researchers have used GO slim to perform tasks in which GO term comparison is required. However, it is evident that while using a subset of GO terms or a reduced version of GO, such as GO slim, to relate genes makes GO terms and annotations easier to work with, valuable information is lost in the simplification. In addition, GO semantic similarity measures can also be used to assess the biological relevance of an interaction network as proteins with similar cellular functions tend to interact in the networks. In fact, they constitute a critical feature for protein analysis and have a dominant performance in discriminating true protein interactions from noise [3, 4].

Broadly speaking, there exist two main classes of GO term similarity approaches, namely, edge- (or path-) and information content-based approaches. The edge-based approach is the oldest approach that was proposed for measuring similarity between terms in a hierarchical semantic structure. In this approach, similarity between two terms is a function of the number of edges (or nodes) on a shortest path between these terms, which is actually the inverse multiplicative of the number of nodes in a shortest path between terms [5]. The logarithm version of this approach was suggested in which the similarity between two terms is given by the negative logarithm of the ratio between the length of the shortest path and twice the maximum depth of the hierarchy under consideration [6]. In this traditional approach, the shorter the length of the shortest path, the more semantically similar the two terms are. It is limited to edge counting and fails to take into account the positions of terms expressing their specificity in the hierarchy. In order to attenuate this shortcoming, some researchers weighted edges by assigning lower weight to edges at the lower level (close to the root) compared to edges at higher level in the hierarchy. However, terms at the same depth do not necessarily have the same specificity, and edges at the same level do not necessarily represent the same semantic distance [7]. Note that throughout this study, the root of the hierarchy is assumed to be located at level 0 and considered to be a reference level.

As the influence of the position of the two terms is essential and must be considered in the computation of similarity between terms, several approaches based on information content (IC) or semantic value of a term have been introduced. The earliest proposal on the IC-based approaches was suggested by Lord et al. [8], who used Resnik's metric [9] to quantify semantic similarity between terms in the GO DAG, which is the information content of the most informative common ancestor (MICA) of these terms; that is, the similarity between two terms is simply the information content of the most specific parent of these terms. This approach has been criticized quantitatively and qualitatively. The quantitative criticisms are results of limitations of the measure to properly capture similarities in the hierarchy under consideration, and this has led to the use of other metrics, such as Lin [10] and Jiang and Conrath [11]. Several other corrections, such as disjunct common ancestor by Couto et al. [12], relevance similarity by Schlicker et al. [13], and information coefficient similarity by Li et al. [14], have been proposed in order to improve existing GO term comparison approaches. The qualitative criticism is due to the shallow annotation dependence when computing the information content values, which leads to serious theoretical validation issues. Indeed, a given term in the GO DAG may have different information content values depending on the corpus used, whereas a term in the GO DAG is expected to have a unique information content value which should not depend on the corpus under consideration.

The issue of the uniqueness of the information content value for a given term can be solved by using the mapping between proteins and the GO annotations provided by the GO annotation (GOA) project [15–17]. However, the fact that IC depends on the annotation statistics related to terms may produce biased IC values since a term can be rarely used, but not necessarily very specific considering its position in the GO DAG. Even though the use of the IC make senses from a probabilistic point of view [7], the shallowness of annotation artifacts will persist when comparing pairs of proteins annotated with few terms [18]. Thus, due to the need for providing an approach that is able to overcome annotation-based issues, approaches depending only on the topology of the GO DAG referred to as topology-based approaches were introduced. Topology-based approaches aim to correct the effect of annotation dependence to provide an effective way to measure similarity between proteins based only on the GO DAG, producing a fixed and well-defined information content for a given GO term independent of the corpus under consideration. These topology-based approaches include the GO-universal metric introduced by Mazandu and Mulder [19] and the Zhang et al. [20] and Wang et al. [21] methods.

Several semantic similarity measures have been introduced and successfully applied to several biomedical applications. These are very often evaluated on the basis of biological relevance, that is, how they capture protein sequence similarity, or how they perform in clustering analysis, and so forth. Theoretical analysis providing a validation of the theories at different levels of descriptions remains to be done. This will provide a basis for exploring their properties to determine whether the measure under consideration is valid, that is, well defined. This study revisits IC-based GO semantic similarity metric approaches that have been proposed and consistently describes them in a unified framework theory to provide a systematic way of deriving all the measures, encapsulating all these approaches, and analyzing them in order to identify their common features. We performed an experimental evaluation of these measures to explore how the information content model used to express the specificity of a term in the GO DAG affects a measure's performance.

## 2. Methods and Materials

This section briefly describes the existing approaches used to compute the information content (IC) or semantic value (SV) of a given term in the hierarchy. We review the theoretical basis of similarity between concepts, infer a unified framework for existing GO term semantic similarity approaches, and predict other possible approaches within this unified framework. Finally, we provide examples of these GO term semantic similarity approaches, illustrating the computation of term semantic similarity values.

### 2.1. Computing Term Information Content

From its conception, term information content (IC) approaches can be divided into two families: annotation and topology-based IC approaches. While topology-based approaches exploit only the intrinsic topology of the GO DAG, the annotation-based approach requires the addition of annotation data for the corpus under consideration. With exception of the topology-based approach proposed by Wang et al., all other approaches compute the IC of terms in a similar way despite their conceptual differences. The IC of the term is given by

(1) $$\begin{array}{c} \text{IC}\left(x\right)=-\ln p\left(x\right). \end{array}$$

In the case of annotation-based approaches, *p*(*x*) is the relative frequency of the term *x* in the protein dataset under consideration, obtained from frequency *f*(*x*) representing the number *𝒜*(*x*) of proteins annotated with the term *x* in the dataset considering the “true-path rule” principle of the GO DAG structure. Thus, this frequency *f*(*x*) is given by

(2) $$\begin{array}{c} f\left(x\right)=\{\begin{array}{cc} 𝒜\left(x\right) & \text{if}\;x\;\text{is}\;\text{a}\;\text{leaf}\\ 𝒜\left(x\right)+\sum_{z\in {𝒞}_{h}\left(x\right)}𝒜\left(z\right) & \text{otherwise}, \end{array} \end{array}$$

where *𝒞*_*h*_(*x*) is the set of GO terms having *x* as a parent, and a leaf is a term that has no child.

In the case of the topology-based approach introduced by Zhang et al., *f*(*x*) is called the count of the term *x*, it depends only on the children of a given GO term and is numerically equal to the sum of counts of all its children. *f*(*x*) is calculated using a recursive formula starting from leaves in the hierarchical structure and given by

(3) $$\begin{array}{c} f\left(x\right)=\{\begin{array}{cc} 1 & \text{if}\;x\;\text{is}\;\text{a}\;\text{leaf}\\ \sum_{z\in {𝒞}_{h}\left(x\right)}f\left(z\right) & \text{otherwise}. \end{array} \end{array}$$

The relative frequency *p*(*x*), called the *D*-value in the case of the topology-based approach used here, is then computed independently for each ontology and given by

(4) $$\begin{array}{c} p\left(x\right)=\frac{f\left(x\right)}{f\left(r\right)}, \end{array}$$

where *f*(*r*) is the frequency (count) of the root term in the ontology under consideration.

In the context of the GO-universal approach, *p*(*x*) is called the topological position characteristic of *x*, recursively obtained using its parents gathered in the set *𝒫*_*x*_ = {*w* : (*w*, *x*) ∈ *ℒ*_GO_} with *ℒ*_GO_ the set of links or associations (*s*, *t*) between a parent *s* and its child *t* in the GO-DAG, and given by

(5) $$\begin{array}{c} p\left(x\right)=\{\begin{array}{cc} 1 & \text{if}\;x\;\text{is}\;\text{a}\;\text{root}\\ \prod_{w\in {𝒫}_{x}}\frac{p\left(w\right)}{\left|{𝒞}_{h}\left(w\right)\right|} & \text{otherwise}, \end{array} \end{array}$$

with |*𝒞*_*h*_(*w*)| as the number of children with term *w* as parent.

Wang introduced a topology-based semantic similarity measure in which the semantic value of a given term *x* is computed using an *S*-value related to the term *x* and given by

(6) $$\begin{array}{c} S_{x}\left(t\right)=\{\begin{array}{cc} 1 & \text{if}\;t=x\\ \max\left\{\omega_{e}*S_{x}\left(t^{\prime }\right):t^{\prime }\in {𝒞}_{h}\left(t\right)\right\} & \text{otherwise}, \end{array} \end{array}$$

with *𝒞*_*h*_(*t*), the set of children of the term *t* and *ω*_*e*_ as the semantic contribution factor for “is_a” and “part_of” relations set to 0.8 and 0.6, respectively. The information content or a semantic value of a term *x* is calculated as follows:

(7) $$\begin{array}{c} {\text{IC}}_{W}\left(x\right)=\sum_{t\in T_{x}}S_{x}\left(t\right), \end{array}$$

where *T*_*x*_ = *T* ∪ {*x*} and *T* denotes the set of ancestors of the term *x*.

### 2.2. Comparing GO Terms

Lin [10] investigated the theoretical basis of similarity and consistently derived the general form of an information-theoretic measure for object similarity. Based on similarity axioms, the similarity measure between two objects *A* and *B*, denoted *𝒮*(*A*, *B*), is viewed as a question of how much information two objects have in common and how much they differ by, given by

(8) $$\begin{array}{c} 𝒮\left(A,B\right)=\frac{\mu \left(A\cap B\right)}{\mu \left(A\cup B\right)}, \end{array}$$

where *μ*(*A*∩*B*) ≥ 0 is the measure of the commonality between *A* and *B*, and *μ*(*A* ∪ *B*) > 0 is a measure of the description of *A* and *B*, with *μ*(*A*∩*B*) ≤ *μ*(*A* ∪ *B*).

#### 2.2.1. Fundamental Formula of GO Term Similarity Metric

In the context of the GO DAG, the similarity measure between terms in the hierarchy is driven by the two functions *μ*_*∞*_, *μ*_1_ : *𝒫*(*𝒩*_GO_) → [0, +*∞*), with *𝒫*(*𝒩*_GO_) as the set of subsets of the set *𝒩*_GO_ of all terms in the hierarchy, measuring the description of subset *T*_*x*_⊆*𝒩*_GO_ induced by a given term *x* ∈ *𝒩*_GO_ and defined as follows:

(9) $$\begin{array}{c} \mu_{\infty }\left(T_{x}\right)=\max\left\{\delta \left(t\right):t\in T_{x}\right\},\\ \mu_{1}\left(T_{x}\right)=\sum_{t\in T_{x}}\delta \left(t\right), \end{array}$$

where *δ*(*t*) is the measure of specificity of the term *t* given by

(10) $$\begin{array}{c} \delta \left(t\right)=\{\begin{array}{cc} S_{x}\left(t\right) & \text{for}\;\text{the}\;\text{Wang}\;\text{et}\;\text{al}.\;\text{approach}\\ \text{IC}\left(t\right) & \text{otherwise}. \end{array} \end{array}$$

For *x* ∈ *𝒩*_GO_, we denote ||*x*||_*∞*_ = *μ*_*∞*_(*T*_*x*_) and ||*x*||_1_ = *μ*_1_(*T*_*x*_), which define the specificity of the term *x* or a distance of the term *x* to the root. Note that ||*x*||_*∞*_ = IC(*x*) and ||*x*||_1_ = SV(*x*) and this “norm” notation is expressly used to emphasize the fact that IC(*x*) or SV(*x*) is in fact the distance or the length from the term *x* to the root term of the hierarchy under consideration.

In all the node-based approaches, the commonality between terms *a* and *b* and their description are summarized in the following formula:

(11) $$\begin{array}{c} \mu \left(T_{a}\cap T_{b}\right)=\epsilon \mu_{p}\left(T_{a}\cap T_{b}\right),\\ \mu \left(T_{a}\cup T_{b}\right)=\alpha \mu_{p}\left(T_{a}\cap T_{b}\right)+\beta \mu_{p}\left(T_{a}\right)+\gamma \mu_{p}\left(T_{b}\right), \end{array}$$

with *p* = 1, *∞*, *ϵ* is the adjustment parameter correcting the overestimation of term commonality with 0 < *ϵ* ≤ 1 and may depend on common ancestors between *a* and *b*. *α*, *β*, *γ* ≥ 0 are three free parameters with *α* + *β* + *γ* ≥ 1. We obviously have *μ*(*T*_*a*_∩*T*_*b*_) ≤ *μ*(*T*_*a*_ ∪ *T*_*b*_). Indeed,

(12) μ(Ta∩Tb)  =ϵμp(Ta∩Tb)≤μp(Ta∩Tb)  ≤(α+β+γ)μp(Ta∩Tb)  =αμp(Ta∩Tb)+βμp(Ta∩Tb)+γμp(Ta∩Tb)  ≤αμp(Ta∩B)+βμp(Ta)+γμp(Tb)  =μ(Ta∪Tb).

It turns out that all the known GO IC-based similarity measures, apart from those related to the Resnik approach, can be retrieved from the following formula:

(13) $$\begin{array}{c} 𝒮\left(a,b\right)=\frac{\epsilon \mu_{p}\left(T_{a}\cap T_{b}\right)}{\alpha \mu_{p}\left(T_{a}\cap T_{b}\right)+\beta \mu_{p}\left(T_{a}\right)+\gamma \mu_{p}\left(T_{b}\right)}. \end{array}$$

Note that unlike the Resnik approach in which the commonality measure is considered as the similarity measure between two terms and whose values may not range between 0 and 1, the general similarity metric formula (13) is normalized; that is, their values range between 0 and 1.

Some studies [4, 22, 23] have normalized the Resnik approach by using either the possible upper bound of IC values [23], referred to as the Nunif strategy, or the highest IC score, referred to as the Nmax strategy, in the ontology under consideration [4, 22]. In this case, the normalized Resnik similarity scores between two terms are given by

(14) $$\begin{array}{c} {𝒮}_{\text{Nunif}}\left(a,b\right)=\frac{\text{IC}\left(c\right)}{\log_{2}N},\\ {𝒮}_{\text{Nmax}}\left(a,b\right)=\frac{\text{IC}\left(c\right)}{{\text{IC}}_{\max}}, \end{array}$$

where *N* is the number of annotated proteins in the corpus under consideration, IC_max_ = max{IC(*t*) : *t* ∈ *𝒩*} with *𝒩* as the set of all terms used in the annotation set for the ontology under consideration, and *c* is the MICA between GO terms *a* and *b*.

**Figure 1:**
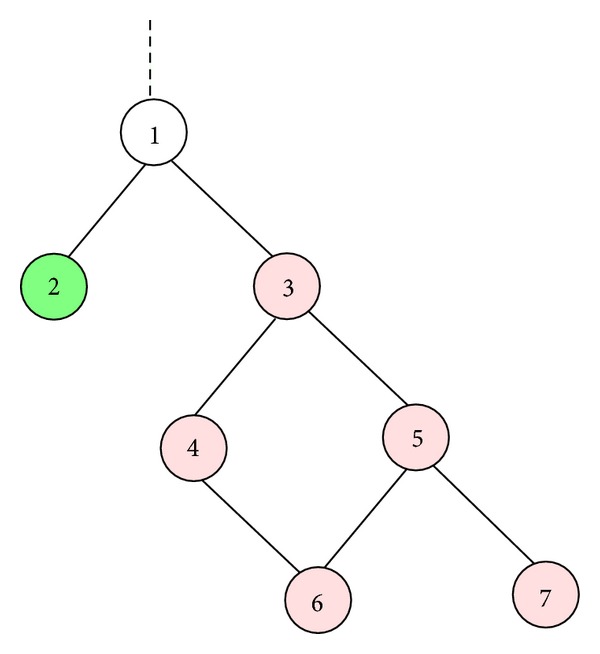
Illustrating the inconsistency of the Resnik approach.

It is worth mentioning that not only does this classical Resnik method not follow the theoretical basis of similarity measurements between concepts, but it is also often inconsistent with the hierarchy under consideration. We illustrate this inconsistency using Figure 1. According to the Resnik approach, the semantic similarity score between nodes 2 and 3 is equal to those between 2 and all descendants of node 3, which is the IC score of node 1. This is not consistent for a hierarchical structure in which a child term is expected to be more specific or to have a higher IC value than its parents. One expects the semantic similarity scores between nodes 2 and all descendants of 3 to be less than that between nodes 2 and 3; that is, node 3 should be more semantically similar to node 2 than to any of its descendants. Here, we suggest using the GO-universal normalization concept, referred to as Nunivers, where the semantic similarity score between terms *a* and *b* is given by

(15) $$\begin{array}{c} {𝒮}_{\text{Nunivers}}\left(a,b\right)=\frac{\text{IC}\left(c\right)}{\max\left\{\text{IC}\left(a\right),\text{IC}\left(b\right)\right\}}. \end{array}$$

#### 2.2.2. Inferring All IC-Based GO Term Similarity Measures

**Figure 2:**
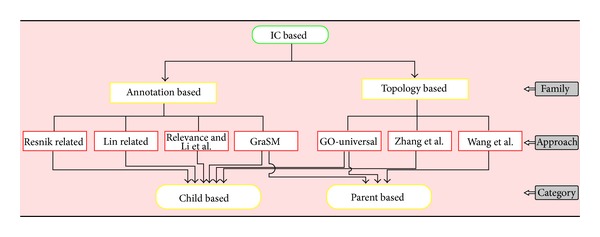
Flowchart of different families, approaches, and categories of existing IC-based GO term semantic similarity measures.

As pointed out previously, we divided the GO term similarity measures into two main families considering how the IC of a term is computed. There are approaches depending only on the intrinsic topology of the GO DAG, referred to as topology-based approaches, and those that also use the frequencies at which terms occur in the corpus under consideration, referred to as annotation-based approaches. Furthermore, depending on the features captured in the computation of the IC of a term or that of similarity measure between two terms under consideration, we have classified the similarity measures into two groups, namely, child- and parent-based approaches. Child-based approaches are those using children term features in their computation, and parent-based approaches use parent term features. Other studies have referred to these approaches as Graph-based approaches. These different approaches are shown in Figure 2, and different parameters for each GO term similarity approach are summarized in Table 1.

All the annotation-based approaches, except the GraSM approach suggested by Couto et al. [12, 24], as well as the topology-based approaches, except the Wang et al. approach, use only the most informative common ancestor (MICA) of the two GO terms *a* and *b*. In this case, the similarity between these two terms is proportional to the information content of the most informative common ancestor between them. They are referred to as MICA approaches. From Table 1, we see that (13) describes all IC-based semantic similarities between terms in a single framework. Even though the value of *α* is zero for the existing approaches, it seems useful to have a general *α* in (13) as this allows us to capture other approaches which have been proposed in the context of path-based approaches. For example by taking *ϵ* = *α* = 1 and *β* = *γ* = 1/2, (13) also describes the Wu and Palmer approach [25] suggested in the context of edge- or path-based approaches. This is given by

(16) $$\begin{array}{c} {𝒮}_{\text{WP}}\left(a,b\right)=\frac{2\times \text{len}\left(r,c\right)}{\text{len}\left(r,a\right)+\text{len}\left(r,b\right)+2\times \text{len}\left(r,c\right)}, \end{array}$$

where *r* is the root of the hierarchy, len(*r*, *c*) is the maximum depth from the root to all common ancestors of *a* and *b*, and len(*r*, *a*) and len(*r*, *b*) are maximum depths from the root to the terms *a* and *b*, respectively. Another path-based similarity measure captured by (13) by taking *ϵ* = *α* = *β* = *γ* = 1 is that used in [26, 27], defined as follows:

(17) $$\begin{array}{c} {𝒮}_{eb}\left(a,b\right)=\frac{\text{len}\left(r,c\right)}{\text{len}\left(r,c\right)+\text{len}\left(c,a\right)+\text{len}\left(c,b\right)}, \end{array}$$

where len(*x*, *y*) is the length in number of edges of the longest distance between terms *x* and *y*.

Furthermore, keeping a general *α* is also very important for theoretical reasons as the formula aims at explaining current and all future possible IC-based semantic similarity measures in the biomedical and bioinformatics fields. Applying the Wang concept on IC, one can define a new range of GO term semantic similarity measures based on the Tversky ratio model [28] using the function *μ*_1_ from (13).

#### 2.2.3. Illustrating Different IC-Based Term Similarity Groups

The graph-based similarity measure (GraSM) approach incorporates the characteristics of the hierarchy by selecting disjunctive common ancestors of the two terms under consideration. Two common ancestors are considered to be disjunctive if there are independent paths from both ancestors to the term. Thus, GraSM computes the commonality of two terms as the average of the information content of their disjunctive common ancestors (DCAs). At the same time, the features of terms through the “true path” rule are captured in the computation of IC of the term. The GO-universal approach uses the richness of the GO DAG structure through the topology position characteristic of the term, which takes into account not only parent features but also children that the term has. These two approaches belong to child- and parent-based categories and are referred to as hybrid approaches. The Wang et al. approach considers only parent term features and is classified as a parent-based approach, while others consider only children term features through the “true path” rule and are classified as child-based approaches.

**Figure 3:**
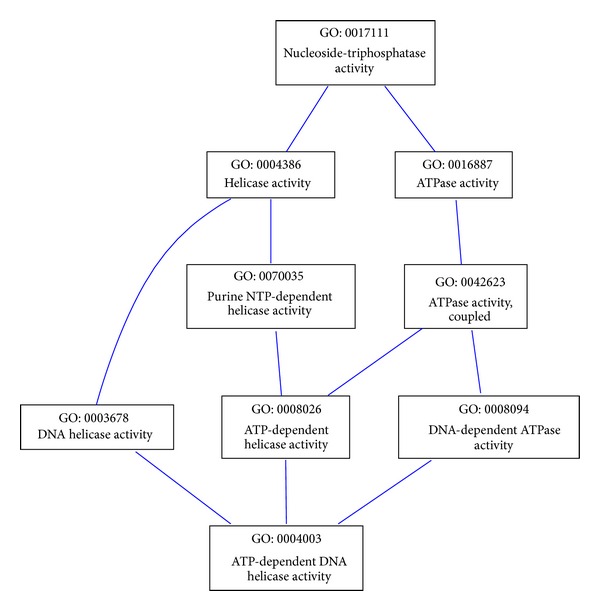
Snapshot of the term GO:0004003 in the molecular function ontology.

We illustrate how the MICA and the DCA approaches work in the snapshot of the GO molecular function ontology shown in Figure 3. This snapshot has been extracted from the sub-GO DAG in the AmiGO browser, using GO term GO:0004003 as a key. To compute the semantic similarity between GO:0008026 (*ATP-dependent helicase activity*) and GO:0003678 (*DNA helicase activity*), the MICA approaches only consider the most informative common ancestor GO:0004386 (*helicase activity*), whereas the DCA approaches also consider the independent path (GO:0008026, GO:00042623, GO:0016887, GO:0017111) from *ATP-dependent helicase activity* to *nucleoside-triphosphatase activity*. Thus, for DCA approaches, the similarity between GO:0008026 and GO:0003678 is proportional to the mean of the information content of their common disjunctive ancestors, namely, GO:0017111 (*nucleoside-triphosphatase activity*) and GO:0004386 (*helicase activity*). This means that the DCA approaches correct the MICA similarity score between two terms only if there are independent paths from common ancestors to the terms under consideration. For example, the similarity score between GO:0070035 (*purine NTP-dependent helicase activity*) and GO:0003678 (*DNA helicase activity*) is proportional only to the information content of GO:0004386 (*helicase activity*), in which case the similarity score is the same as that produced by the MICA approaches.

Note that the so-called Jiang and Conrath approach has not yet been mentioned. Jiang and Conrath [11] suggested an approach to compute the distance between terms in a given hierarchy using the idea of the distance to the root of the hierarchy to define the distance between two terms *a* and *b* as the sum of their distances to their MICA. So, let *c* denote the MICA of terms *a* and *b*, we have

(18) dJC(a,b)=(||a||∞−||c||∞)+(||b||∞−||c||∞)=IC(a)+IC(b)−2×IC(c).

As the distance between two terms can be converted to their semantic similarity measure, Couto et al. [24] infer a semantic similarity measure from the Jiang and Conrath distance as follows:

(19) $$\begin{array}{c} {𝒮}_{\text{JC}}\left(a,b\right)=\frac{1}{1+d_{\text{JC}}\left(a,b\right)}. \end{array}$$

Several other researchers [22, 23] have attempted to infer a semantic similarity from the Jiang and Conrath distance using the following generic formula:

(20) $$\begin{array}{c} {𝒮}_{\text{JC}}\left(a,b\right)=1-D_{\text{JC}}\left(a,b\right), \end{array}$$

provided that *D*_JC_(*a*, *b*) is a normalized value of *d*_JC_(*a*, *b*), that is, ranges between 0 and 1. For example, Couto et al. [22] used the following normalization scheme:

(21) $$\begin{array}{c} D_{\text{JC}}\left(a,b\right)=\min\left\{1,\frac{d_{\text{JC}}\left(a,b\right)}{\text{IC}\left(t_{0}\right)}\right\}, \end{array}$$

where *t*_0_ is a term that only occurs once in the corpus under consideration. Pesquita et al. [23] defined the normalized distance as follows:

(22) $$\begin{array}{c} D_{\text{JC}}\left(a,b\right)=\frac{{\text{IC}}_{u}\left(a\right)+{\text{IC}}_{u}\left(b\right)}{2}-{\text{IC}}_{u}\left(c\right), \end{array}$$

where IC_*u*_(*x*) is the uniform IC of *x*, given by

(23) $$\begin{array}{c} {\text{IC}}_{u}\left(x\right)=\frac{\text{IC}\left(x\right)}{\log_{2}N} \end{array}$$

with *N* as the number of annotated proteins in the corpus under consideration.

Here, we note that by using canonical normalization of this distance, where the original distance is divided by the maximum possible distance between terms IC(*a*) + IC(*b*), we obtain the Lin approach; that is,

(24) $$\begin{array}{c} {𝒮}_{L}\left(a,b\right)=1-\frac{d_{\text{JC}}\left(a,b\right)}{\text{IC}\left(a\right)+\text{IC}\left(b\right)}. \end{array}$$

In other words, *d*_JC_ is simply the nonnormalized distance derived from the Lin semantic similarity approach. The other normalization schemes were unable to improve the performance of semantic similarity inferred from the Jiang and Conrath distance [23]. This is why we are not referring to Jiang and Conrath, as the best semantic similarity measure inferred from this distance is Lin's approach.

### 2.3. Evaluating Protein Functional Similarity

A protein can carry out several molecular functions or participate in several biological processes occurring in different subcellular components, in which case, several GO terms are needed to annotate this protein in a given ontology. For two annotated proteins or sets of GO terms, semantic similarities between GO terms of these proteins or in these sets can be combined to produce semantic or functional similarity between these proteins or sets of GO terms. Four different term semantic similarity combinations are used to retrieve functional similarity scores, including average (Avg) [8], maximum (MAX) [29], best match average (BMA) [19, 23], and average best matches (ABMs) [4, 21]. Note that these four approaches have been used in the context of annotation-based semantic similarity measures. For topology-based semantic similarity measures, each scheme has provided its approach for computing protein functional similarity scores. The GO-universal measure uses the BMA approach, ABM was used in the Wang et al. measure, and the Zhang et al. measure has proposed context dependent methods, but the authors initially suggested using the Avg scheme.

## 3. Results and Discussion

We have derived a unified mathematical framework for all IC-based GO semantic similarity approaches for annotation- and topology-based families and compared these approaches based on theoretical formula parameters. Here, we perform an experimental evaluation of different correction factors suggested in the context of annotation-based approaches and assess the impact of different normalization models proposed in the context of the Resnik approach compared to the normalization idea used in the GO-universal metric. Finally, we compare different topology-based approaches and parent- versus child-based approaches.

We used protein pairs from the Collaborative Evaluation of Semantic Similarity Measures (CESSM) online tool [30] at http://xldb.di.fc.ul.pt/tools/cessm/. The CESSM tool is an online tool that enables the comparison of new measures against previously published annotation IC-based GO similarity measures in terms of Pearson's correlation measures with sequence, Pfam domain, and Enzyme Commission (EC) similarity as well as resolution and assessing how sensitive the approach is to differences in the annotations based on the sequence similarity scores [23]. GO annotations of these proteins were obtained from the GOA-UniProtKB project, release 2013-01 of January 9, 2013, with GO biological process (BP), molecular function (MF), and cellular component (CC) terms from the GO database version 1.3499.

### 3.1. Effects of Normalization Models

As pointed out previously, several studies have attempted to normalize the Resnik similarity measure in the context of annotation-based approaches. For example, Couto et al. [22, 24] suggested the idea of using the highest information content value in the hierarchy as described in (21). This strategy has also been used by Jain and Bader [4] to normalize the similarity scores between terms. Pesquita et al. [23] uniformize similarity measures to provide scores that range between 0 and 1. These normalization strategies have been an issue for the performance of the similarity metric induced by the Jiang and Conrath distance approach. Here, we are using the normalization idea used in the GO-universal approach to normalize Resnik's similarity measure and compare its performance to the previous ones. Note that we use all types of GO evidence codes when assessing different measures, and the best match average (BMA) approach was used for computing functional similarity scores between proteins for all annotation-based approaches as it has been suggested to be better than the average (Avg) or maximum (Max) approaches not only empirically, but also from a biological point of view [23, 31].

Results are shown in Tables 2, 3, and 4 for the GO BP, MF and CC ontologies, respectively. For the BP ontology, the normalization strategy using the GO-universal idea (Nunivers strategy) outperforms other normalization techniques by consistently showing one of the highest correlations with sequence, Pfam and EC similarity, except for resolution, where the strategy of using the highest IC value (Nmax strategy) performs better. For the MF ontology, the normalization strategy using the Nunivers strategy produces lower Pearson's correlation with PFAM and sequence similarity compared to the other normalization strategies, but overall, it outperforms them in terms of EC similarity. For the CC ontology, Nmax and Nunif strategies perform better than the Nunivers strategy in terms of EC, Pfam, and sequence similarity, but the Nunivers strategy consistently outperforms Nmax and Nunif strategies in terms of resolution. These observations suggest that using the Nunivers strategy for normalizing GO term semantic similarity scores is more appropriate than the Nmax strategy considering the inconsistency of these Resnik-related approaches as explained previously (see Section 2.2.1). The Nmax and uniformizing (Nunif) strategies perform equally, producing approximately equal Pearson's correlation, and the former strategy produces the highest resolution. Thus, it is beneficial to use the Nmax strategy for normalizing GO term semantic similarity scores if one has to choose between the two strategies.

### 3.2. Effect of Correction Factor Models

We first look at the results produced by enhancements suggested by relevance similarity due to Schlicker et al. [13] and the information coefficient idea of Li et al. [14] in order to improve the Lin approach. The information coefficient idea consistently outperforms the relevance approach, showing highest correlation with sequence, Pfam, and EC similarity, as well as for resolution. Note that for the CC ontology, no enhancement strategy suggested could improve the performance of the original Lin approach in terms of resolution.

The GraSM approach has significantly improved the performance of annotation-based approaches; however, finding the disjunctive common ancestors (DCA) between two GO terms makes the GraSM approach computationally unattractive, especially for a dense DAG, and this computational complexity is not always proportional to the improvement in performance. Thus, we assess the impact of using all informative common ancestors (ICA) shared between two distinct terms under consideration, instead of using only the disjunctive common ancestors. This is referred to as an eXtended GraSM (XGraSM) approach, in which the correction factor is computed as in the GraSM approach but considering all informative common ancestors (ICAs) shared by distinct terms under consideration. Indeed, the XGraSM approach consistently improves the performance of annotation-based approaches and outperforms the GraSM approaches. It is worth mentioning that GraSM and XGraSM approaches are hybrid semantic similarity measures in which features of parent and child terms are taken into account. They are shown to perform better than other enhancement strategies and to improve these semantic similarity measures. This observation was also emphasized by Mazandu and Mulder [19], suggesting that for improving a GO term semantic similarity measure, it should take into account features of parents and children of terms.

GraSM, XGraSM, and other enhancement strategies suggest that the performance of annotation-based approaches can be improved. However, the fact that the conception of their term IC relies on the annotation data, specifically on the annotation statistics related to terms, biases the scores produced. This is a serious drawback to the annotation based methods and will remain an unsolvable issue for these approaches. From its conception, a GO term occurring less often in the corpus under consideration will be more specific, independent of its position in the GO-DAG. In fact, some terms are more often used to annotate proteins in the corpus than others, possibly because of the research focus of these terms, and this is not related to their specificity in the GO-DAG translated by their IC scores. In addition, these approaches are unable to score an orphan term's specificity. An orphan term refers to a GO term that has not been used in the corpus, and these terms are present in the GO DAG even if protein annotations are retrieved from the GOA-UniProtKB project, which is the largest set of annotations. Indeed, only 13633, 6670, and 1975 GO terms from the BP, MF and cellular component (CC) ontologies, respectively, have been directly used to annotate proteins. Through the “true path” rule, additional GO terms are indirectly used as protein annotations, and there are total of 16625 out of 24492 BP active terms, 7009 out of 9532 MF active terms, and 2139 out of 3129 CC active terms. This indicates that many GO terms are orphans in the GO DAG and their specificity scores cannot be quantified.

### 3.3. Evaluating Topology-Based Approaches

Looking at different Pearson's correlations for the topology-based approaches, the GO-universal approach shows the highest correlation with sequence and Pfam similarity for the BP ontology, while Zhang et al. is highest for EC similarity. In the case of the MF ontology, GO-universal generally performs well, producing comparable Pearson's correlation for EC similarity with the Zhang et al. approach and consistently outperforms the Wang et al. and Zhang et al. approaches in terms of sequence and Pfam similarity, as well as resolution. Thus, again the GO-universal approach achieves overall best performance for these topology-based approaches for BP and MF ontologies. It also outperforms previous annotation-based approaches (excluding the new XGraSM related approaches) in terms of Pfam and sequence similarity for the BP ontology. For the CC ontology, the Wang et al. approach performs better in terms of EC, Pfam and sequence similarity, but the GO-universal approach consistently outperforms all approaches, including annotation-based approaches, in terms of resolution. Note that each topology-based approach is implemented with its associated functional similarity measure as suggested by the authors of the approach, except for the Zhang et al. approach, which is implemented with the average best matches (ABMs) as it has been shown to improve the performance of this approach [4].

It has been suggested that a given similarity approach relying on the intrinsic topology of the hierarchical structure should consider both GO term parents and children in its conception [19]. It is more likely that missing overall information of a term's children in the IC conception of the Wang et al. approach and missing overall information of parents in the IC conception of the Zhang et al. approach have negatively impacted these methods. On the other hand, even though annotation-based approaches have been improved through the XGraSM approach, their dependence on annotation data constitutes an unsolvable drawback of these approaches. Furthermore, as enhancing any GO measures should start from the conception of GO term IC, the GO-universal metric, which includes parent and child information in its conception, is possibly a route toward the solution to the issue of scoring a term's specificity in the GO DAG.

## 4. Conclusions

In this work, we have set up a unified theory in a well-defined mathematical framework describing all existing IC-based GO semantic similarity measures. This scheme with one correction factor and three free parameters explains current and should explain all future possible IC-based semantic similarity measures in the biomedical and bioinformatics fields. We have performed experimental evaluations of different correction factors suggested in the context of annotation-based approaches and assess the impact of different normalization models proposed in the context of the Resnik approach, as well as different topology-based approaches through analysis using the Collaborative Evaluation of Semantic Similarity Measures (CESSM) online tool. Results show that to perform well, a given GO-semantic similarity measure should consider a term's parent and children information in its conception. As the fundamental measure in the GO-DAG is the term IC, a plausible solution to the issue of scoring a term's specificity is thus that the IC should consider the term's parents and children information in its conception. This suggests that the GO-universal metric is possibly an appropriate solution to the issue of scoring term specificity in the GO DAG.

## Figures and Tables

**Table 1:** Comparison of different IC-based approach parameters. In the GO term semantic similarity approaches, c is the MICA between GO terms a and b, and *n* is the number disjunctive common ancestors between terms a and b, the *n*th being the MICA between a and b.

Family	Approach	Parameters				*𝒮*(*a*, *b*)
		*p*	*α*	*β*, *γ*	*ϵ*	
Annotation	Lin	∞	0	$\frac{1}{2},\frac{1}{2}$	1	$\frac{2\times \text{IC}(c)}{\text{IC}(a)+\text{IC}(b)}$
	Relevance	∞	0	$\frac{1}{2},\frac{1}{2}$	1 − *e* ^−IC(*c*)^	$\frac{\epsilon \times 2\times \text{IC}(c)}{\text{IC}(a)+\text{IC}(b)}$
	Li et al.	∞	0	$\frac{1}{2},\frac{1}{2}$	1 − (1+IC(*c*))^−1^	$\frac{\epsilon \times 2\times \text{IC}(c)}{\text{IC}(a)+\text{IC}(b)}$
	GraSM	∞	0	$\frac{1}{2},\frac{1}{2}$	$\frac{1}{n}\left(1+\overset{n-1}{\underset{j=1}{\sum }}\frac{\text{IC}\left(t_{j}\right)}{\text{IC}\left(c\right)}\right)$	$\frac{\epsilon \times 2\times \text{IC}(c)}{\text{IC}(a)+\text{IC}(b)}$

	Wang et al.	1	0	1,1	1	$\underset{t\in A\cap B}{\sum }\frac{S_{a}(t)+S_{b}(t)}{\text{SV}(a)+\text{SV}(b)}$
Topology	Zhang et al.	∞	0	1,1	1	$\frac{2\times \text{IC}(c)}{\text{IC}(a)+\text{IC}(b)}$
	GO-Universal	∞	0	1,0 or 0,1	1	$\frac{\text{IC}(c)}{\max\left\{\text{IC}\left(a\right),\text{IC}\left(b\right)\right\}}$

**Table 2:** Comparison of performance of different approaches for GO BP ontology. This comparison is done using Pearson's correlation with enzyme commission (EC), Pfam and sequence similarity, and resolution. Results are obtained from the CESSM online tool. The best scores among each group are in bold, and Nmax, Nunif, and Nunivers are suffixes indicating different IC normalization strategies, namely, the highest IC value, uniform, and GO-universal strategies, respectively.

Family	Approach	Similarity measure correlation			Resolution
		EC	PFAM	Seq Sim	
Annotation	Resnik-Nmax	0.41166	0.29151	0.54563	**0.55874**
	Resnik-Nunif	0.41166	0.29151	0.54563	0.49265
	Nunivers	**0.48967**	**0.41280**	**0.62349**	0.48490
	Lin	0.48032	0.38900	0.57956	0.43343
	Li et al.	**0.49531**	**0.42010**	**0.62173**	**0.49017**
	Relevance	0.48188	0.38682	0.57550	0.43823
	GraSM-Lin	0.48673	**0.45470**	0.61739	0.51701
	GraSM-Nmax	0.44826	0.35941	0.63497	0.54996
	GraSM-Nunif	0.44826	0.35941	0.63497	0.48491
	GraSM-Nunivers	**0.49301**	0.44158	**0.65671**	**0.92975**
	XGraSM-Lin	0.39811	**0.49859**	0.68669	**0.92067**
	XGraSM-Nmax	0.45493	0.37152	0.69892	0.53910
	XGraSM-Nunif	0.45493	0.37152	0.69892	0.47533
	XGraSM-Nunivers	**0.49782**	0.45220	**0.70732**	0.91425

Topology	Wang et al.	0.45451	0.47867	0.65214	**0.91475**
	Zhang et al.	**0.47888**	0.45527	0.61862	0.44350
	GO-universal	0.45958	**0.48175**	**0.68953**	0.43772

**Table 3:** Comparison of performance of different approaches for GO MF ontology. This comparison is done using Pearson's correlation with enzyme commission (EC), Pfam and sequence similarity, and resolution. Results are obtained from the CESSM online tool. The best scores among each group are in bold, and Nmax, Nunif, and Nunivers are suffixes indicating different IC normalization strategies, namely, the highest IC value, uniform, and GO-universal strategies, respectively.

Family	Approach	Similarity measure correlation			Resolution
		EC	PFAM	Seq Sim	
Annotation	Resnik-Nmax	0.64381	**0.49101**	**0.59663**	**0.55309**
	Resnik-Nunif	0.64381	0.49101	0.59662	0.28872
	Nunivers	**0.70697**	0.47693	0.40945	0.41671
	Lin	0.67404	0.42844	0.36060	0.36583
	Li et al.	**0.70287**	**0.46309**	**0.38823**	**0.44311**
	Relevance	0.67618	0.42112	0.35081	0.39798
	GraSM-Lin	0.68125	0.44009	0.37243	0.38321
	GraSM-Nmax	0.65180	**0.49844**	**0.60405**	0.37213
	GraSM-Nunif	0.65180	0.49844	0.60405	0.28859
	GraSM-Nunivers	**0.71257**	0.48638	0.41889	**0.43191**
	XGraSM-Lin	0.70480	0.53732	0.47682	0.43007
	XGraSM-Nmax	0.67136	**0.58792**	**0.70911**	0.36781
	XGraSM-Nunif	0.67136	0.58792	0.70911	0.28524
	XGraSM-Nunivers	**0.71965**	0.55251	0.48988	**0.47064**

Topology	Wang et al.	0.64327	0.46102	0.37272	0.34873
	Zhang et al.	**0.68296**	0.43453	0.35581	0.38646
	GO-universal	0.67661	**0.47000**	**0.38190**	**0.43772**

**Table 4:** Comparison of performance of different approaches for GO CC ontology. This comparison is done using Pearson's correlation with enzyme commission (EC), Pfam and sequence similarity, and resolution. Results are obtained from the CESSM online tool. The best scores among each group are in bold, and Nmax, Nunif, and Nunivers are suffixes indicating different IC normalization strategies, namely, the highest IC value, uniform, and GO-universal strategies, respectively.

Family	Approach	Similarity measure correlation			Resolution
		EC	PFAM	Seq Sim	
Annotation	Resnik-Nmax	0.34355	**0.43796**	**0.55437**	0.40935
	Resnik-Nunif	0.34355	0.43796	0.55437	0.33651
	Nunivers	0.32079	0.40931	0.53991	**0.95526**
	Lin	0.29912	0.38851	0.4998	**0.96253**
	Li et al.	**0.32110**	**0.40980**	**0.54062**	0.95511
	Relevance	0.30183	0.39132	0.50435	0.96131
	GraSM-Lin	0.30463	0.38749	0.51142	**0.95896**
	GraSM-Nmax	0.36341	**0.45946**	**0.60546**	0.40677
	GraSM-Nunif	0.36341	0.45946	0.60546	0.33439
	GraSM-Nunivers	**0.32473**	0.41170	0.55626	0.95247
	XGraSM-Lin	0.30812	0.39642	0.57390	**0.95087**
	XGraSM-Nmax	**0.37079**	**0.47364**	**0.68564**	0.39428
	XGraSM-Nunif	0.37079	0.47364	0.68564	0.32412
	XGraSM-Nunivers	0.32451	0.41225	0.59762	0.94673

Topology	Wang et al.	**0.34404**	**0.39297**	**0.61451**	0.94019
	Zhang et al.	0.00477	0.00246	0.00188	0.36749
	GO-universal	0.15787	0.19982	0.13119	**1.00000**
